# Chemical Instruction

**Published:** 1850-08

**Authors:** 


### CHEMICAL INSTRUCTION.

The attention of medical students will be arrested by the advertisement of Professor B. Silliman, Jr., on the cover of this Journal. We are much gratified in the proposed arrangements of that able and excellent teacher, and we feel assured that he will give more than an ordinary impulse to the science of chemistry in the West. It is a science of the highest interest, and its principles enter largely into almost every department of human pursuits. If an intimate knowledge of chemistry is essential to the physician, it is scarcely less so, to the farmer, and we hope that some of the latter class will embrace the opportunity, offered by Professor Silliman, for giving their sons an insight into the science of chemistry. The young farmer who may acquire a knowledge of the science, and who applies it well to farming, will possess many advantages over a blundering routinist, who cannot imagine why particular fields of his farm never can be made to yield a remunerative crop of wheat, while others that look like them, always succeed. A knowledge of the constituent elements of wheat, and an analysis of soils will explain the whole mystery, and make a very simple affair of it.

In view of these, and of kindred matters, we hail with more than ordinary satisfaction the proposed measures of Professor Silliman. Of his eminent qualifications, as a teacher of the natural sciences, we need not say a word. His reputation is widely known, and we have never known a teacher who won a more enviable position among students than he did last winter. B.

